# Electrospun Poly(ester-Urethane)- and Poly(ester-Urethane-Urea) Fleeces as Promising Tissue Engineering Scaffolds for Adipose-Derived Stem Cells

**DOI:** 10.1371/journal.pone.0090676

**Published:** 2014-03-04

**Authors:** Alfred Gugerell, Johanna Kober, Thorsten Laube, Torsten Walter, Sylvia Nürnberger, Elke Grönniger, Simone Brönneke, Ralf Wyrwa, Matthias Schnabelrauch, Maike Keck

**Affiliations:** 1 Division of Plastic and Reconstructive Surgery, Department of Surgery, Medical University of Vienna, Vienna, Austria; 2 Biomaterials Department, INNOVENT e. V., Jena, Germany; 3 Ludwig Boltzmann Institute for Experimental and Clinical Traumatology, Austrian Cluster for Tissue Regeneration, Vienna, Austria; 4 Department of Traumatology, Medical University of Vienna, Vienna, Austria; 5 Research Department Applied Skin Biology, Beiersdorf AG, Hamburg, Germany; Instituto de Engenharia Biomédica, University of Porto, Portugal

## Abstract

An irreversible loss of subcutaneous adipose tissue in patients after tumor removal or deep dermal burns makes soft tissue engineering one of the most important challenges in biomedical research. The ideal scaffold for adipose tissue engineering has yet not been identified though biodegradable polymers gained an increasing interest during the last years. In the present study we synthesized two novel biodegradable polymers, poly(ε-caprolactone-co-urethane-co-urea) (PEUU) and poly[(L-lactide-co-ε-caprolactone)-co-(L-lysine ethyl ester diisocyanate)-block-oligo(ethylene glycol)-urethane] (PEU), containing different types of hydrolytically cleavable bondings. Solutions of the polymers at appropriate concentrations were used to fabricate fleeces by electrospinning. Ultrastructure, tensile properties, and degradation of the produced fleeces were evaluated. Adipose-derived stem cells (ASCs) were seeded on fleeces and morphology, viability, proliferation and differentiation were assessed. The biomaterials show fine micro- and nanostructures composed of fibers with diameters of about 0.5 to 1.3 µm. PEUU fleeces were more elastic, which might be favourable in soft tissue engineering, and degraded significantly slower compared to PEU. ASCs were able to adhere, proliferate and differentiate on both scaffolds. Morphology of the cells was slightly better on PEUU than on PEU showing a more physiological appearance. ASCs differentiated into the adipogenic lineage. Gene analysis of differentiated ASCs showed typical expression of adipogenetic markers such as PPARgamma and FABP4. Based on these results, PEUU and PEU meshes show a promising potential as scaffold materials in adipose tissue engineering.

## Introduction

A high incidence of soft tissue damage due to trauma or tumor removal on the one hand and limitations in reconstructing these defects on the other hand, asks for new solutions such as tissue engineering approaches. Tissue engineering works with biomimetic methods combining material engineering with life science. It requires an engineered biodegradable and highly biocompatible scaffold which can be used as vehicle for (stem) cells, growth factors, drugs, genes or other bioactive factors. This material should serve as first artificial matrix in tissue defects supporting invading cells to produce a new extracellular matrix and stimulating them to proliferate and form the new functional tissue. Due to recent progress in the development of new biomaterials and improved scaffold processing techniques over the last decades, new promising scaffold materials can be fabricated and offered to patients [1]-[4]. However, the main focus in research and clinical application lies in dermal and epidermal substitutes [5], whereas the development of a subcutaneous replacement (hypodermis) is often neglected. This is in contrast to its overall importance: the hypodermis serves not only as energy storage but also defines the shape of the body and is well known as an endocrine organ. Generation of materials with mechanical and biological properties comparable to native adipose tissue is still a challenge for researchers active in this field [6], [7]. A new, appropriate biomaterial should not only be stable for several weeks to serve as a framework for invading cells, but should also be biodegradable and hold a certain thickness and elasticity to provide plasticity as filler and shock protection. Certain characteristics such as tensibility or micro- and nano-structure are important to imitate the natural extracellular matrix.

During the last years, besides hydrogel formation [8], [9], electrospinning has gained much interest as processing technique providing promising scaffold structures in soft tissue engineering [10]-[14]. This technique not only offers a high flexibility in material selection including synthetic and also natural polymers, but also provides nano- or microstructured three-dimensional scaffolds that resemble the extracellular matrix and support the mechanical stability of tissue. Such scaffolds allow cells to detach and communicate with each other. In principle, they are able to support cell differentiation, extracellular matrix generation and vascularization [15]. In addition to biocompatibility and mechanical stability, an adequate porous structure of the matrix seems to be a crucial factor for cell differentiation and integration. Controlling the fabrication parameters of the electrospinning process to optimize fiber diameter, pore structure, as well as mesh density and thickness with regard to cell cultivation requirements is therefore an important task [16], [17].

A variety of novel biocompatible copolymers have been electrospun to fabricate nanofibrous scaffolds for biomedical applications with different success. But not only the material compositions but also the fabrication process can be manipulated in order to change fiber diameter, morphology and scaffold porosity [18]. Electrospun fibrous scaffolds can be prepared with a high degree of control over their structure, creating highly porous meshes of ultrafine fibers that resemble the ECM topography [19]. The fibrillar structure can enhance cell attachment, proliferation and colony-forming capacity of stem cells in vitro in comparison with non-fibrillar tropocollagen layers [19].

Both biodegradable biopolymers and synthetic polymers can be processed by electrospinning [20]. Naturally derived materials such as collagen [21], derivatives of hyaluronic acid [22], matrigel [23], and fibrin [24] have been intensively studied as scaffolds in adipose tissue engineering. Collagen which is prevalent in the native extracellular matrix (ECM) is known to promote adipose tissue development in vivo. Unfortunately if seeded with cells collagen devices often show contraction and rapidly degrade in vivo [25]. Similar to collagen fibrin gels are able to support adipogenesis in vivo, but like collagen the material has a high degradation rate and has not been studied extensively as 3D, porous scaffold [26], [27]. Hyaluronic acid which is also a component of many ECM is a readily water-soluble and degradable polymer. It therefore has to be chemically modified for use as scaffold materials. In several attempts porous sponges of hyaluronic acid esters possessing a slower degradation rate have been used in adipose tissue engineering. Human preadipocytes cultured on those scaffolds have been shown to differentiate into adipocytes in culture but their properties in vivo remain to be investigated. Several studies have demonstrated that Matrigel, a commercially available protein mixture containing ECM components like laminin is highly adipogenic in vivo when injected in mice together with growth factors like bFGF [26]. Unfortunately Matrigel is not a viable option for clinical use due to its tumor cell origin. To date no satisfying results have yet been published neither in vitro nor in vivo. We can only speculate why there is such a lack of data. Maybe because the need for a hypodermis is not as urgent as for epidermis and dermis. Besides, a final construct of generated adipose tissue will need vascularization, which makes any approach a lot more difficult.

As an advantage of synthetic polymers their mechanical properties and biodegradation behavior can be tailored over a wide range [28]. Therefore this class of materials is able to meet biological and medical requirements in tissue engineering. Especially biodegradable polymers gained an increasing interest in the last decade. Recent developments in this group of materials are focused on polyesters like polycaprolactone (PCL) [29] or poly(lactic acid) (PLA) [30]–[33]. Because PLA homopolymers are rather brittle materials and PCL homopolymers due to their low glass transition temperatures show only limited mechanical stability at body temperature and, in addition, due to their hydrophobic nature a low rate of degradation, often copolymers of PLA and PCL or even terpolymers containing further monomers are used as scaffold materials [34]–[37]. Besides that, polyurethanes (PU) are a very promising group of polymeric materials. They often show excellent elasticity and can be designed-to-degrade at the same time, which makes them a promising alternative to poly(hydroxy acids) homopolymers or their copolyesters [38], [39]. This is especially true if instead of toxicologically problematic aromatic isocyanates those, derived from natural sources, are used in polyurethane synthesis like L-lysine ethylester diisocyanate (LDI) [40]–[43]. In fact, the combination of polyesters with polyurethanes in related poly(ester-urethane) block co- or terpolymers should lead to (thermoplastic) polymers with excellent physical properties including elasticity and sufficient mechanical strength combined with a more variable degradation behaviour. Concerning the mechanical properties, in the present case a potentially useful scaffold materials should show viscoeleastic or viscoplastic bahaviour and a minimum tensile strength of about 5 MPa.

Adipose-derived stem cells (ASCs) are immature precursor cells located between mature adipocytes in adipose tissue [44]. These cells can serve as an ideal autologous cell source for adipose tissue engineering approaches, since they are more resistant to mechanical damage and ischemia than mature adipocytes [45]. Adipose-derived stem cells can be harvested during liposuction or resection of adipose tissue and have been shown to proliferate rapidly and differentiate into bone, adipogenic and chondrogenic lineage both in vitro and in vivo [46]–[48].

In the present study we synthesized two novel urethane-based polymers, a poly(ester-urethane) (PEU) and a poly(ester-urethane-urea) (PEUU). Micro-structured fleeces with adjusted morphological characteristics suitable for soft tissue engineering were fabricated from these polymers by electrospinning. We hypothesized that due to the different chemical composition and structure of the polymers the produced scaffolds strongly differ in their mechanical properties and their degradation behaviour. The main goal of this study was to elucidate the cell compatibility as well as ASCs viability, proliferation and differentiation on the two scaffolds evaluating their potential as a framework in adipose tissue engineering

The scope of this work is on the tailoring of synthetic biodegradable polymers matching the specific requirements in adipose tissue engineering. Due to the mechanical properties diverging from those of common well-known, degradable polymers, these materials have the potential to gain attraction also in other fields of soft tissue regeneration.

## Materials and Methods

### Ethics Statement

This study has been approved by the ethics committee of the Medical University of Vienna and the General Hospital Vienna (EK no. 957/2011). All subjects enrolled in this research have given written informed consent. Fat tissue was obtained from 10 donors undergoing abdominoplasty.

### Materials and General Procedures

Dichloromethane (DCM), cyclohexane, heptane and toluene were purchased from Fisher Scientific (Schwerte, Germany) and used without further purification. Acetone, chloroform (CHCl_3_) and methanol (MeOH) used for electrospinning and analytical purposes (HPLC grade) were obtained from VWR International (Darmstadt, Germany). Hexafluoroisopropanol (HFIP) was purchased from Apollo Scientific Ltd. (Stockport, U.K.). L-lactide was purchased by PURAC Biomaterials (Gorinchem, Netherlands). ε-Caprolactone (Sigma-Aldrich, Taufkirchen, Germany) and 2,6-diisocyanato methyl caproate (L-lysine ethyl ester diisocyanate) (Infine Chemicals, Shanghai, China) were purified by vacuum distillation. Polyethylene glycol (M_w_  =  400 g mol^−1^, PEG 400) and N-methylpyrrolidone (NMP) were purchased from Sigma Aldrich. Poly(L-lactide-co-D,L-lactide) (70%/30% (w/w), M_w_  =  1.35×10^6 ^g mol^−1^, PLLA) was purchased from Evonik Röhm (Darmstadt, Germany).

### Polymer syntheses

Representative synthesis procedures for the poly(ester-urethane) and the poly(ester-urethane-urea) materials are as follows:

Poly[(L-lactide-co-ε-caprolactone)-co-(L-lysine ethyl ester diisocyanate)-*block*-oligo(ethylene glycol)-urethane] (PEU)

Octanediol-bis(L-lactide-co-ε-caprolactone) (LLA-CL)

A mixture of 1,8-octanediol (1.0 g, 6.84 mmol), L-lactide (19.7 g, 136.78 mmol), and 16.55 µl stannous octoate (dissolved in 94 µl toluene) was stirred under nitrogen at 150°C. After 45 minuntes further 16.55 µl stannous octoate (dissolved in 94 µl toluene) was added. The mixture was then stirred for 75 minutes at this temperature followed by an addition of ε-caprolactone (58.04 ml, 547.11 mmol) and 49.9 µl stannous octoate (dissolved in 280 µl toluene). Stirring was continued at 150°C for 1 hour, followed by an addition of 49.9 µl stannous octoate. Then the solution was stirred for 3 hours, cooled to room temperature and dissolved in 75 ml dichloromethane. The solution was filtrated and the crude product was precipitated into 1000 ml cold heptane. Finally, the isolated LLA-CL was dried in vacuum at room temperature to constant weight (76.9 g).

NMR: 1.4–1.25 (m; 4.6 H); 1.65–1.40 (m; 11.8 H); 2.25 (t; 3.9 H); 4.01 (t; 4 H); 5.15–5.00 (m; 1 H)

Poly[(L-lactide-co-ε-caprolactone)-co-(L-lysine-ethyl ester-diisocyanato)urethane] prepolymer (LLA-CL-LDI)

In the next step, L-lysine ethyl ester diisocyanate (LDI) (12.77 ml, 63.24 mmol) was combined with *LLA-CL* (76.9 g) under nitrogen and reacted at 60°C with stirring for 4 hours. After cooling to room temperature the reaction mixture was dissolved in 80 ml dichloromethane, filtrated and precipitated twice into 1000 ml cold cyclohexane. Finally, the isolated isocyanate-terminated poly(L-lactide-co-ε-caprolactone) prepolymer (LLA-CL-LDI) was dried in vacuum at room temperature to constant weight (71.3 g).

Poly[(L-lactide-co-ε-caprolactone)-co-(L-lysine ethyl ester diisocyanato)-block-oligo(ethylene glycol)urethane] (PEU)

In the last step LLA-CL-LDI (71.3 g) was dissolved in N-methylpyrrolidone (300 ml) and heated to 60°C. PEG 400 (3.01 g, 4.48 mmol) was dissolved in 30 ml NMP and added dropwise. The reaction mixture was stirred overnight, cooled to room temperature and precipitated into 3000 ml of cold water. The crude product was dried in vacuum at room temperature, then dissolved in 100 ml dichloromethane, filtrated and precipitated into 1500 ml cold petrol ether. Finally, the isolated poly(ester-urethane) PEU was dried in vacuum at room temperature to constant weight (65.2 g).

Poly(ε-caprolactone-co-urethane-co-urea) (PEUU)

Octanediol-bis(ε-caprolactone) (CL)

A mixture of 1,8-octanediol (0.5 g, 3.42 mmol), ε-caprolactone (39.02 ml, 342.0 mmol), and 31.6 µl stannous octoate (dissolved in 180 µl toluene) was stirred under nitrogen at 150°C. After 30 and 90 minutes additional 15.8 µl stannous octoate (dissolved in 90 µl toluene) was added. The mixture was stirred 4 h overall, then cooled to room temperature and dissolved in 75 ml dichloromethane. The solution was filtrated and the crude product was precipitated into 1000 ml cold heptane. Finally, the isolated oligomer (CL) was dried in vacuum at room temperature to constant weight (39.9 g, 3.45 mmol according to NMR).

NMR: 1.24–1.40 (m; 50.5 H); 1.54–1.65 (m; 100.2 H); 2.25 (t; 50 H); 3.57 (t; 1 H); 4.00 (t; 49.9 H)

Poly[ε-caprolactone-co-(L-lysine ethyl ester diisocyanato)urethane] prepolymer (CL-LDI)

In the next step, LDI (6.82 ml, 33.76 mmol) was combined with CL (39.9 g, 3.45 mmol) under nitrogen and reacted at 60°C with stirring for 4 hours. After cooling to room temperature the reaction mixture was dissolved in 80 ml dichloromethane, filtrated and precipitated twice into 1000 ml cold cyclohexane. Finally, the isolated isocyanate-terminated polylactone prepolymer (CL-LDI) was dried in vacuum at room temperature to constant weight (37.0 g).

Poly(ε-caprolactone-urethane-urea) (PEUU)

In the last step CL-LDI (37.0 g) was dissolved in THF (40 ml) and LDI (495 µl, 2.45 mmol) were added and mixed thoroughly followed by an addition of DABCO-solution (2.96 ml, 2.8 M). The solvent was vaporized at 40°C. The crude product dissolved in 100 ml dichlormethane, filtrated and precipitated into 1500 ml cold petrol ether. Finally, the isolated poly(ester-urethane-urea) PEUU was dried in vacuum at room temperature to constant weight (33.2 g).

### Polymer structure analytics

Molecular weights (M_n_ and M_w_) and polydispersity indices (PDI) were determined with respect to polystyrene standards by gel permeation chromatography (GPC). The measurements were performed on a set of Shimadzu apparatuses (Shimadzu Deutschland, Duisburg, Germany) using chloroform as eluent. All samples were analysed at room temperature. Chloroform (Fisher Scientific, Germany, stabilised with 1% amylene) was used as eluent, delivered at a flow rate of 1.0 ml min^−1^. The samples were dissolved in chloroform at a concentration of 5 mg ml^−1^. The injection volume was 100 µl. As pre-column a PSS-SDV (100 Å, 8,0×50 mm) and as column PSS-SDV (100 Å, 8,0×300 mm), PSS-SDV (1000 Å, 8,0×300 mm) and PSS-SDV (100000 Å, 8,0×300 mm) were used. As detector a RID 10A (Shimadzu Deutschland) was used.

^1^H- and ^13^C- nuclear magnetic resonance (NMR) was used to characterize the chemical structures and compositions of the synthesized copolymer. The spectra were recorded on a Bruker DRX 400 spectrometer (Bruker BioSpin, Rheinstetten, Germany), using tetramethylsilane as an internal reference and CDCl_3_ as solvent. To determine the monomer ratio or the chain length of the polymers we used the NMR. In case of polycaprolactone polymers the chain length was determined by comparison of the ^1^H-NMR signals at 3.57 ppm being the C**H**_2_-OH signal of the last caprolactone unit in the chain with the signal at 2.25 ppm, being the CO-C**H**_2_-CH_2_-signal. In case of lactide-caprolactone copolymer the monomer ratio was determined by comparison of the ^1^H-NMR signal at 5.15–5.00 ppm, being the CH-proton of lactide, with the signal at 2.25 ppm, being the CO-C**H**_2_-CH_2_-signal of the caprolactone monomer.

### Fabrication of foils and compact samples

Foils were fabricated by evaporation of solvent. 400 mg of the polymers were dissolved in 20 ml dichloromethane and poured into dishes (5.5 cm in diameter). The dishes were kept at room temperature for at least 3 days followed by two days at 40°C. The foils were cut into stripes of 50×5 mm^2^. Compact samples (height 8 mm, diameter 8 mm) were fabricated by melting the polymers at 100–150°C in a silicone mould.

### Fabrication of electrospun non-woven fleeces

A computer-aided electrospinning machine developed at Erich Huber GmbH (Gerlinden, Germany) in collaboration with INNOVENT e. V. (Jena, Germany) and recently commercialized under the trade name E-Spintronic (Erich Huber GmbH, Gerlinden, Germany) was used for the fabrication of electrospun non-woven fleeces. This machine enables the defined adjustment of major spinning parameters and a high process reproducibility. Electrospinning conditions similar to those that have been previously described [10], [49] were used. A stainless-steel straight-end hollow needle of diameter of 0.4 mm was used as nozzle. A glass mirror of 2.5 mm thickness (35×35 cm^2^) was used as counter electrode for collecting the electrospun non-woven fibers. The distance between the needle tip and the collector was maintained at 19–22 cm. The voltage was adjusted to 18–28 kV. The polymers dissolved in suitable solvent and appropriate concentration were fed at a constant rate of 1.5 ml h^−1^ through the syringe to the needle tip resulting in the formation of fibers with diameters of about 0.5 to 1.3 µm. The dimension of the obtained electrospun fleeces was approximately 60 cm^2^_._

### Investigation on material properties

#### Mechanical Properties

Young’s-modulus and tensile strength of foils were determined with a Texture Analyser TA-XT2i (Stable Micro Systems, Godalming, U.K.) with a 5 kg measuring head to characterize the mechanical properties of the polymers. Tensile tests were performed after DIN EN ISO 1798. For each material tested 5 samples were produced. Electrospun fleeces were cut into stripes (1×5 cm^2^) and fixed at 5 kN wedge action grips of an Instron universal testing machine (model 4301, Norwood, MA, USA). Materials were stretched with a rate of 20 mm min^−1^ until the stripe ripped, distance and force were measured. Experiments were performed five times at minimum.

#### Thermal Properties

Glass transition temperatures of polymers PEUU and PEU were measured by DMA with a Perkin Elmer DMA7E instrument. Glass transition temperatures of polymer PLLA was measured by DCS with a Perkin Elmer DSC7 instrument.

#### Matrix Characterization

For cell experiments, fleeces were clamped into cell crowns (scaffdex, Sigma-Aldrich, St. Louis, MO, USA) to provide an even, tensed surface. Then they were incubated in 70% ethanol for 30 minutes, air-dried and washed once with PBS before cell seeding.

#### Scanning Electron Microscopy (SEM)

Adipose-derived stem cells (ASCs) were seeded on fleeces in 24 well cell crowns (6×10^4^ cells). Fleeces (with and without cells) were fixed in fixing solution containing 0.1 M sodium cacodylate and 2.5% glutaraldehyde. Subsequently, fleeces were washed (0.1 M sodium cacodylate without glutaraldehyde), dehydrated with 2,2-dimethoxypropane and dried with hexamethyldisilazane. Samples were sputter coated with palladium gold in an Emitech (Molfetta, Italy) sputter coater SC7620 and analyzed in a SEM Jeol 6510 (Jeol Ltd, Tokyo, Japan).

#### Polymer and fleece Degradation

Compact samples of both polymers PEU and PEUU were incubated in Sörensen phosphate buffer (pH 7.4) at 37°C for 4 weeks, rinsed, dried for 1 week in vacuum and weighted. Samples were covered with fresh buffer and procedure was repeated (total storage time 44 weeks).

Furthermore, fleece samples made from PEU and PLLA were stored in simulated body fluid medium (SBF) at 37°C and released free L-lactate was monitored over 12 weeks with the ENZYTEC D-/L-lactic acid assay (R-Biopharm, Darmstadt, Germany). For comparison an elevated degradation test (refluxing of the non-woven PEU samples for 24 hours in 1 N NaOH solution) was carried out measuring the liberated L-lactate in the same manner.

### Biological characterization

#### ASC Isolation and Cultivation

Minced adipose tissue was washed in phosphate buffered saline (PBS, PAA Laboratories GmbH) and digested with 2 mg/ml collagenase type IV in Hanks’ buffered salt solution (HBSS, both Sigma-Aldrich, St. Louis, MO, USA) for 1 h at 37°C with constant shaking. Cells were filtered through cotton gauze and centrifuged for 5 minutes at 380 g. Red blood cells in the stromal vascular fraction were lysed in 2 ml Red Blood Cell Lysing Buffer (Sigma-Aldrich) and incubated on ice for 8 minutes. Cold medium was added and suspension was filtered through a 70 µm cell filter. Cells were centrifuged for 5 minutes at 380 g and cell pellet was re-suspended in DMEM (PAA Laboratories GmbH), supplemented with 100 units ml^−1^ penicillin, 100 µg ml^−1^ streptomycin (both Life Technologies Ltd, Paisley, UK), and 10% fetal calf serum (Hyclone, Fisher Scientific GmbH, Schwerte, Germany).

For experiments, cells were counted, seeded on scaffolds and cultivated at 37°C in humidified atmosphere with 5% CO_2_.

#### Staining of Live and Dead Cells

Cells were grown on top of fleeces for 48 hours in 24 well cell crowns (6×10^4^ cells). For staining of live and dead cells they were incubated with fluorescein diacetate and propidium iodide fluorescent dye solution (Molecular Probes, Inc., Eugene, OR, USA) according to the manufactureŕs protocol. Microscopy was done on an AxioImager microscope (Zeiss, Jena, Germany).

#### Cell Morphology

Fleece preparation and cell seeding was conducted as described above. Cells were grown on fleeces for 48 hours. After fixation in formalin and permeabilization, cells were stained with 10 µg ml^−1^ TRITC-phalloidin/PBS (Sigma-Aldrich, St. Louis, MO, USA) and 5 µg ml^−1^ 4′,6-diamidino-2-phenylindol (DAPI; SERVA Electrophoresis GmbH, Heidelberg, Germany) under light protection. Microscopic analysis was done on an AxioImager microscope (Zeiss, Jena, Germany).

#### MTT Assay/cell proliferation

Viability of cells was further measured using a CellTiter96 non-radioactive proliferation Assay (Promega Corporation, Madison, WI). Therefore, cells were seeded on fleeces in 24 well cell crowns (6×10^4^ cells). On day one, three and seven cell viability was evaluated according to manufactureŕs protocol. Therefore, 15 µl dye solution was added to 100 µl medium, after two hours of incubation 100 µl stop solution was added and incubated for one hour in the dark. Fleeces were gently shaken to homogenize medium and substrate, 50 µl of the supernatant was transferred into a 96 well plate in triplicates and absorbance was measured on a Wallac 1420 VICTOR2 plate reader (PerkinElmer, Waltham, MA, USA).

#### Differentiation

ASCs were seeded on fleeces in 24 well cell crowns (6×10^4^ cells) for 48 hours for proliferation. To induce adipocyte differentiation, cells were incubated for three days in Preadipocyte Differentiation Medium (PromoCell GmbH, Heidelberg, Germany). Afterwards cells were incubated in Adipocyte Nutrition Medium (PromoCell GmbH, Heidelberg, Germany) without IBMX, medium was changed every third day. Differentiation was evaluated on day 21. For adipored staining, viable cells were washed once with PBS and incubated with AdipoRed Assay Reagent (Lonza, Walkersville, MD, USA) according to manufacturer’s protocol. After 15 minutes, cells were rinsed with PBS and fixed with 4% formalin. Cells were analyzed using an AxioImager microscope (Zeiss, Jena, Germany).

#### Preparation of Polymer-Coated Cell Culture Plates

24 well plates (Becton & Dickinson, Franklin Lakes, NJ, USA) were coated with PEUU and PEU as followed: Each polymer was dissolved in hexafluoroisopropanol to gain a 1% (w/w) solution. 140 µL of this polymer solution was used to coat one well. The excessive solution was removed and the polymer-coated cell culture plates were dried under a fume hood over night. Next day, plates were stored in a cell culture incubator (37°C, 5% CO_2_, 90% humidity) to use.

#### Gene Expression Analysis

ASCs offered by Zen-Bio were used as recommended by the supplier (Lonza, Verviers, Belgium). Cells were seeded in the polymer-coated 24 wells or uncoated control wells (6×10^4^ cells) and cultivated in basal growth medium containing 10% fetal calf serum, L-glutamine and GA-1000 (PBM-2, Lonza, Verviers, Belgium) at 5% CO_2_ and 37°C. To differentiate the cells into adipocytes insulin, dexamethasone, indomethacin and isobutylmethylxanthine (PBM-2, Lonza, Verviers, Belgium) were added to the medium as recommended by the supplier. After cultivating ASCs in differentiation medium, cells were harvested and RNA of ASCs, differentiated for seven days, was isolated using the RNeasy Kit whereas RNA of ASCs, differentiated for 14 days, was isolated using the RNeasy Lipid Tissue Kit (both Qiagen, Hilden, Germany) according to the manufacture’s procedure. Afterwards, RNA was transcribed into cDNA using the High-Capacity cDNA Reverse Transcription Mix (Applied Biosystems, Life Technologies Ltd, Paisley, UK). To analyze gene expression Real-Time TaqMan-PCR was performed using FAM labled primers and the 7900 HT Fast and Sequence Detection System (Applied Biosystems, Darmstadt, Germany). The following PCR conditions were used: 50°C for 2 minutes, 94.5°C for 10 minutes followed by 40 cycles at 97°C for 30 sec and 59.7°C for 1 minute. Data were analyzed using the Sequence detector software supplied with the 7900 Sequence Detector and RQ Manager.

Expression levels were calculated by the 2^−ΔΔCt^-method, whereby GAPDH was used as endogenous reference and undifferentiated cells (donor #1) were used as calibrator and therefore were set as 1.

### Statistics

Data are presented as mean ± SD of at least three independent experiments. Statistical analysis was performed with software SPSS Statistics 19 or Microsoft Excel 2010. Statistical comparisons for all experimental settings were based on two sample t-test, ANOVA or General Linear Model using Tukey’s test with p < 0.05 considered as significant.

## Results

### Polymer Synthesis and Characterization

**Figure 1:**
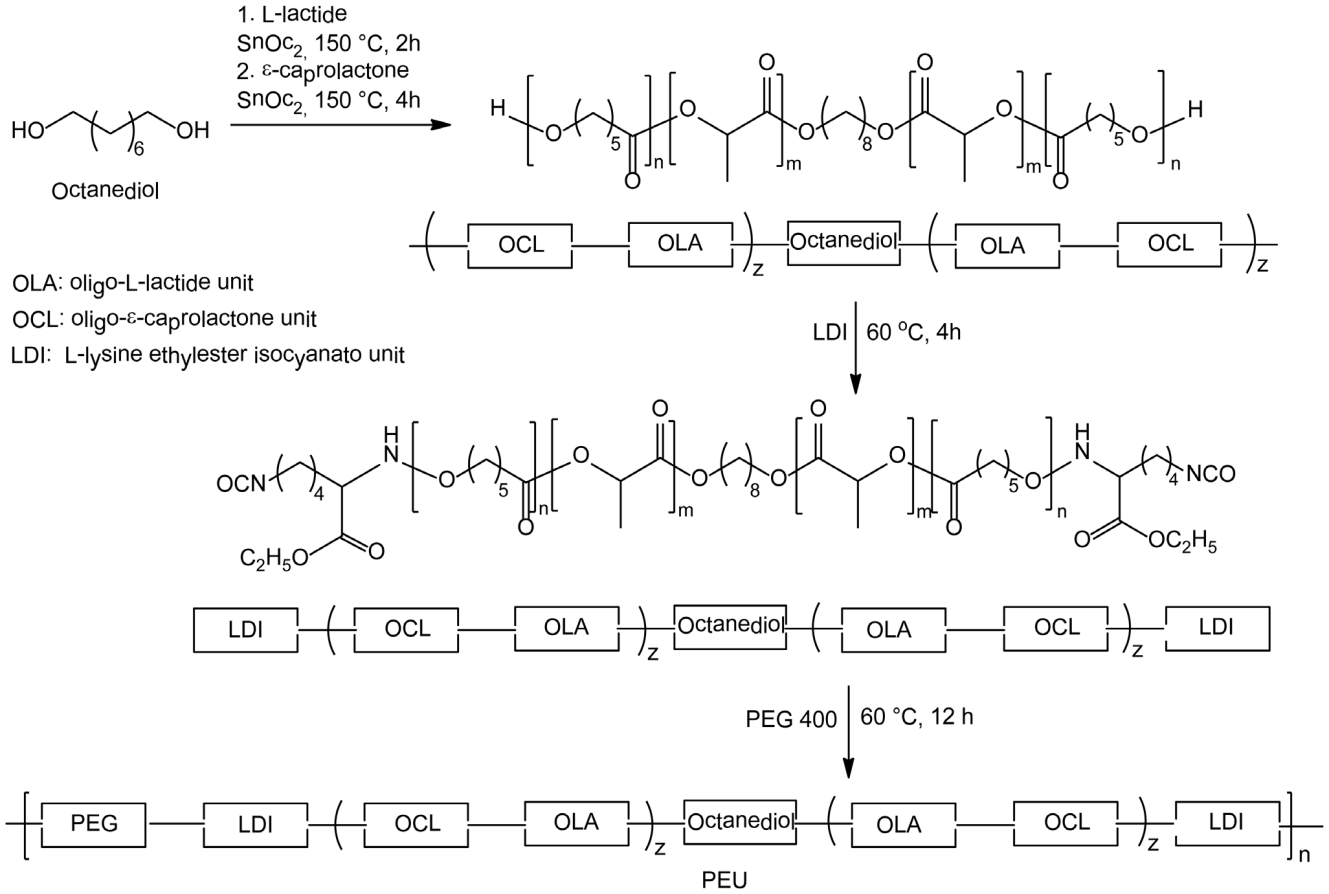
Schematic composition of copolymer PEU.

**Figure 2:**
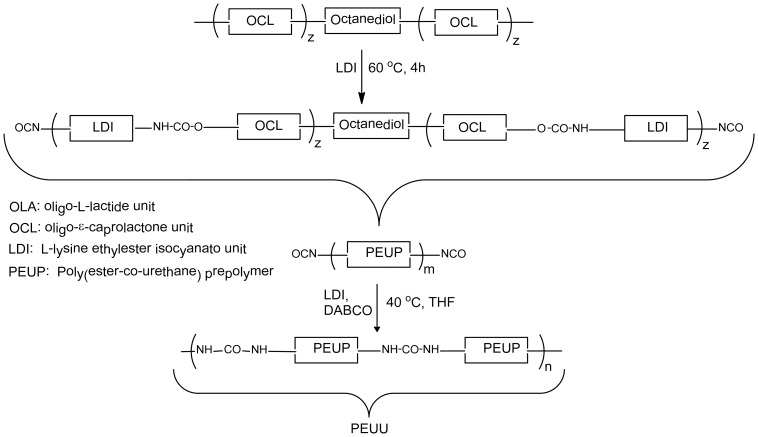
Schematic composition of copolymer PEUU.

Two novel ε-caprolactone-containing polyurethane-type polymers, a poly(ester-urethane) and a poly(ester-urethane-urea) multiblock copolymer, respectively, have been synthesized in a multistep procedure adapting previously reported procedures for the synthesis of polylactones and lysine ethylester based polyurethanes [50], [51]. In the first step oligolactones have been prepared by conventional ring-opening polymerization of ε-caprolactone or L-lactide/ ε-caprolactone. These oligomers have been end-capped with L-lysine ethyl ester diisocyanate (LDI). The resulting reactive prepolymers were treated with PEG 400 to afford the L-lactide- ε-caprolactone containing poly(ester-urethane), PEU, and with LDI in the presence of DABCO as catalyst to obtain the ε-caprolactone based poly(ester-urethane-urea), PEUU, respectively. Figures 1 and 2 schematically illustrate the synthesis strategy and the monomer composition of the prepared PEU and PEUU multiblock polymers.

The polymers and their intermediate prepolymers have been structurally characterized in the usual way by FT-IR spectroscopy, ^1^H- and ^13^C-NMR spectroscopy. Their molecular weights and polydispersity indices have be determined by GPC (Table 1).

**Table 1:** Molecular weights (M_n_, M_w_, M_z_) and polydispersity indices of prepared oligomers and polymers determined by GPC

Sample	Molecular Weight			Dispersity Index
	M_n_	M_w_	M_z_	PDI
CL	22,713	29,607	37,634	1.30
CL-LDI	27,306	38,936	55,006	1.43
PEUU	141,050	495,975	2,397,908	3.52
LLA-CL	22,222	32,933	45,681	1.48
LLA-CL-LDI	24,053	36,286	51,417	1.51
PEU	56,591	108,329	204,829	1.91

**Figure 3:**
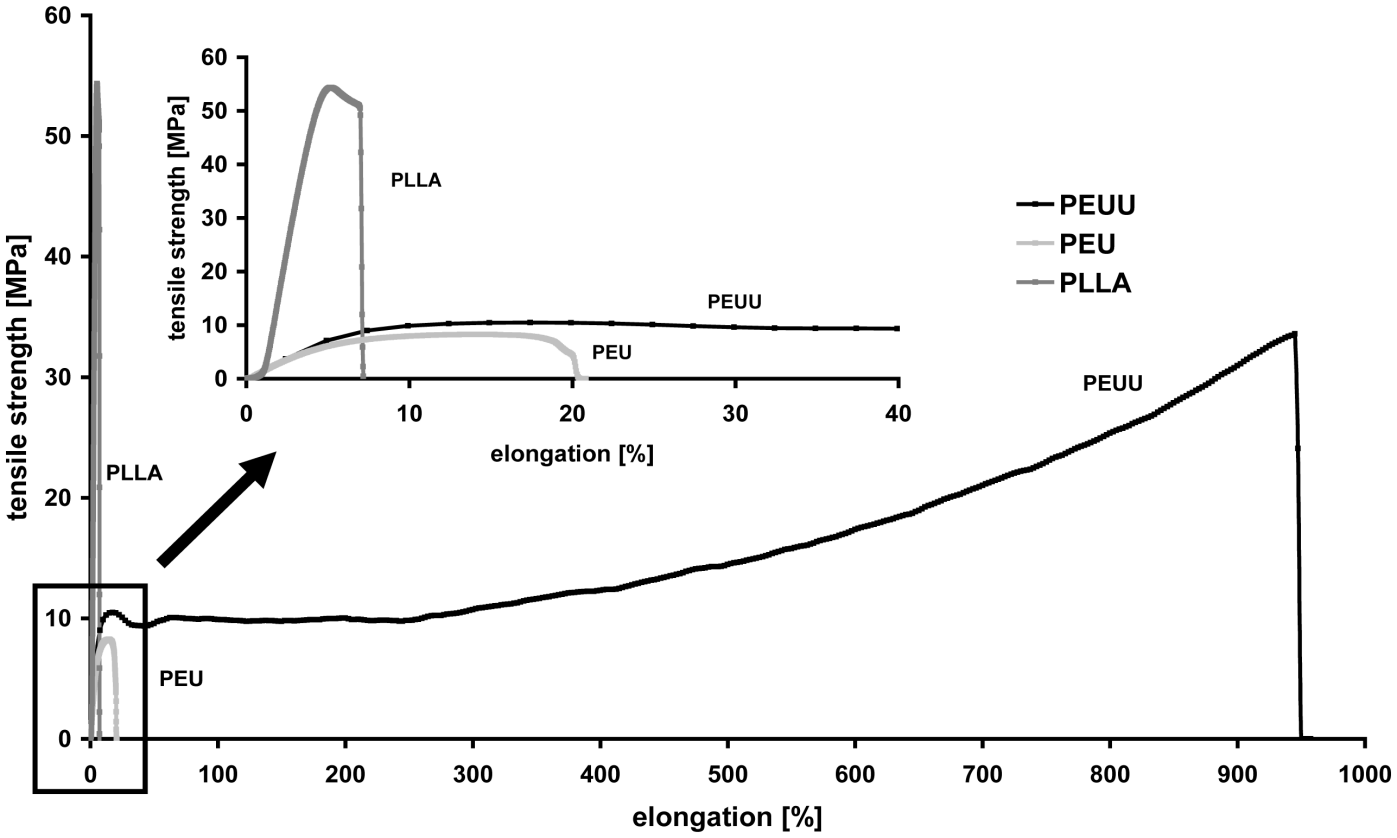
Stress-Strain curves of PEU and PEUU foils in comparison to conventional PLLA foils.

The mechanical properties of the novel polymers were determined using polymer foils. The values for the tensile strength, Young’s modulus, and maximum elongation of the synthesized polymers PEUU and PEU can be found in Table 2. For comparison the corresponding values for a commercially available PLLA are given. The stress-strain curves of the polymers are shown in Figure 3. Young’s modulus was determined at the elastic deformation area at 2–10% elongation. It becomes obvious that the mechanical properties of the ε-caprolactone-containing copolymers remarkably differ from those of PLLA. Due to the presence of the *ε*-caprolactone soft segment, PEU and PEUU have much lower Young’s modulus compared to PLLA. PEUU shows a quite different mechanical behavior in comparison to both the other polymers with regard to elongation prior break. After a steep course of the curve at the beginning in the stress-strain diagram, similar to the course of the other polymers, at higher stress this polymer exhibits plastic deformation resulting in a high maximum elongation at break (Figure 3). A possible explanation of this behavior could be the presence of a large ε-caprolactone block within the PEUU polymer in contrast to the L-lactide/ε-caprolactone copolymer unit in PEU. A similar behaviour has been reported for copoylmers with high *ε*-caprolactone contents [52]. However with regard to the complex structure of PEUU further investigations are necessary to fully understand this phenomenon.

**Table 2:** Mechanical properties (tensile strength, Young’s modulus, and maximum elongation) and glass transition temperatures (T_g_) of synthesized polymers (PEUU, PEU) in comparison to PLLA.

Polymer	Mechanical properties			T_g_
	Tensile strength [MPa]	Young’s modulus [MPa ]	Max. elongation [%]	Peakmaximum tan delta [°C]
PEUU	37.16 (±5,37)	184 (± 8)	1072.39 (±103.88)	–24 a
PEU	7.88 (±0.66)	131 (± 77)	14,64 (±0.73)	8 a
PLLA	50.62 (±3.40)	1393 (± 272)	5.65 (±0.60)	59 ^b^

a peakmaximum tan delta [°C] (measured by DMA); ^b^ half C_p_ (measured by DSC).

### Characteristics of Electrospun Polymer Fleeces

Both polymers could be transformed into electrospun fleeces. For comparative studies, fleeces were also fabricated from commercially available PLLA using the same processing conditions. Electrospinning parameters for PLLA, PEUU and PEU are listed in Table 3.

**Table 3:** Electrospinning parameters and resulting fiber diameters of the electrospun non-woven biomaterials (flow rate for all materials: 1.5 ml h^−1^)

Polymer	Solvent(s) (v/v)	Concentration in % (w/w)	Air humidity [%]	Temperature [°C]	Voltage [kV]	Electrode distance [cm]	Fiber diameter [µm]
PLLA	CHCl_3_/MeOH (3:1)	3	25–36	23–30	20	19–22	0.56–0.89
PEUU	CHCl_3_	4	27–40	21–25	20–22	22	1.02–1.28
PEU	acetone	23	31–55	23–29	18–24	20	0.65–1.14

Fiber diameters were measured on the basis of light microscopy images. It was found that fibers in PEUU are thicker and have a wider range of different microstructures compared to the fibers of biomaterials PLLA and PEU.

### Surface Morphology and Characterization

**Figure 4:**
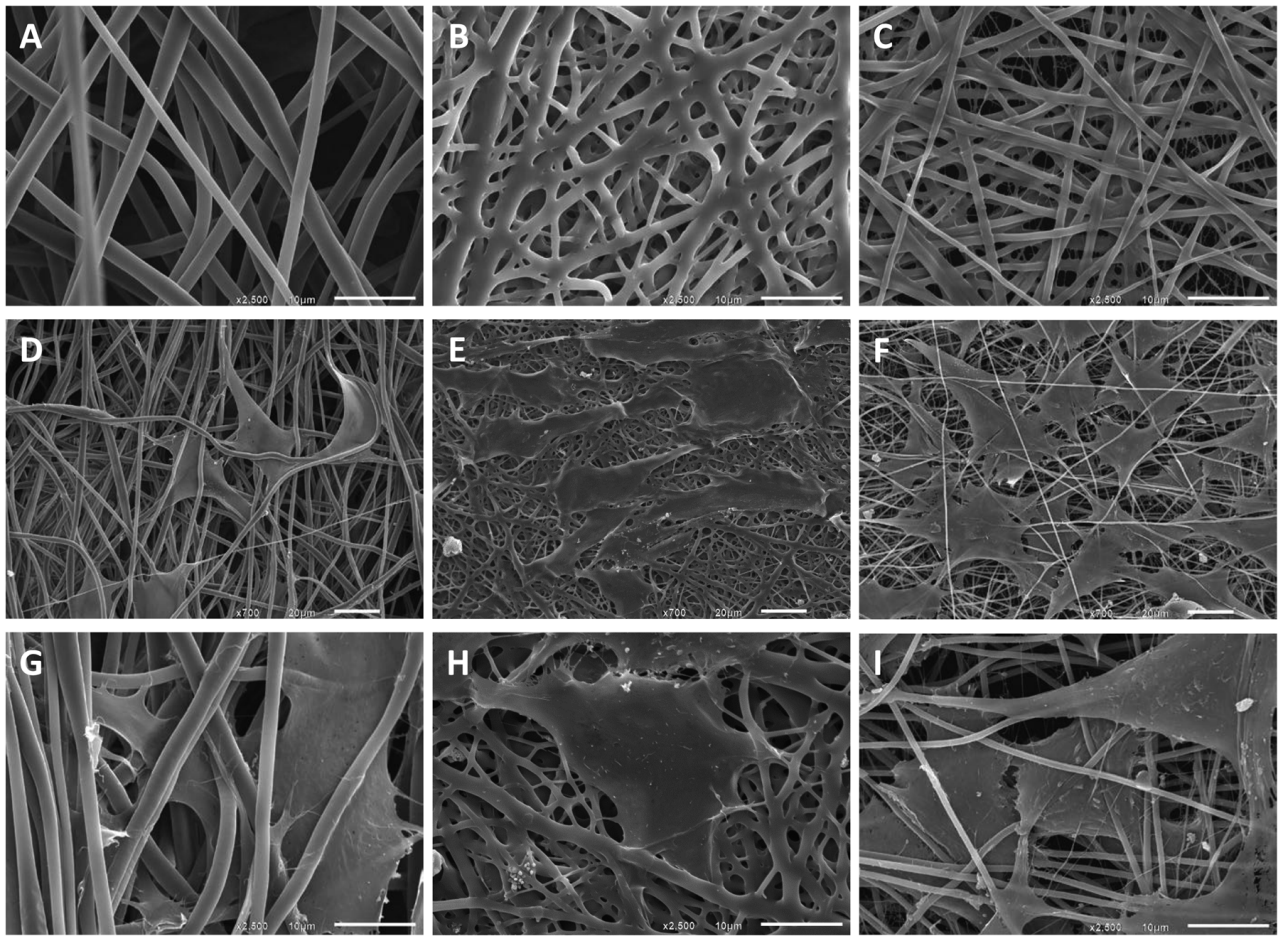
Ultrastructure analyses. SEM images of electrospun materials PEUU (A, D, G), PEU (B, E, H) and PLLA (C, F, I). Adipose derived stem cells were cultivated on top of the materials for 48 hours (D-I). Bars represent 10 µm (A, B, C), 20 µm (D, E, F), or 10 µm (G, H, I), respectively.

The ultrastructure of the electrospun materials was observed by SEM (Figure 4, A-C; materials seeded with adipose derived stem cells for 48 hours: D-I). The thickest fibers occurred in PEUU (1.02–1.28 µm) whereas the fiber thickness of PLLA and PEU were comparable (0.65–1.14 µm) (Table 3). The latter one is woven tighter and has more branches.

**Figure 5:**
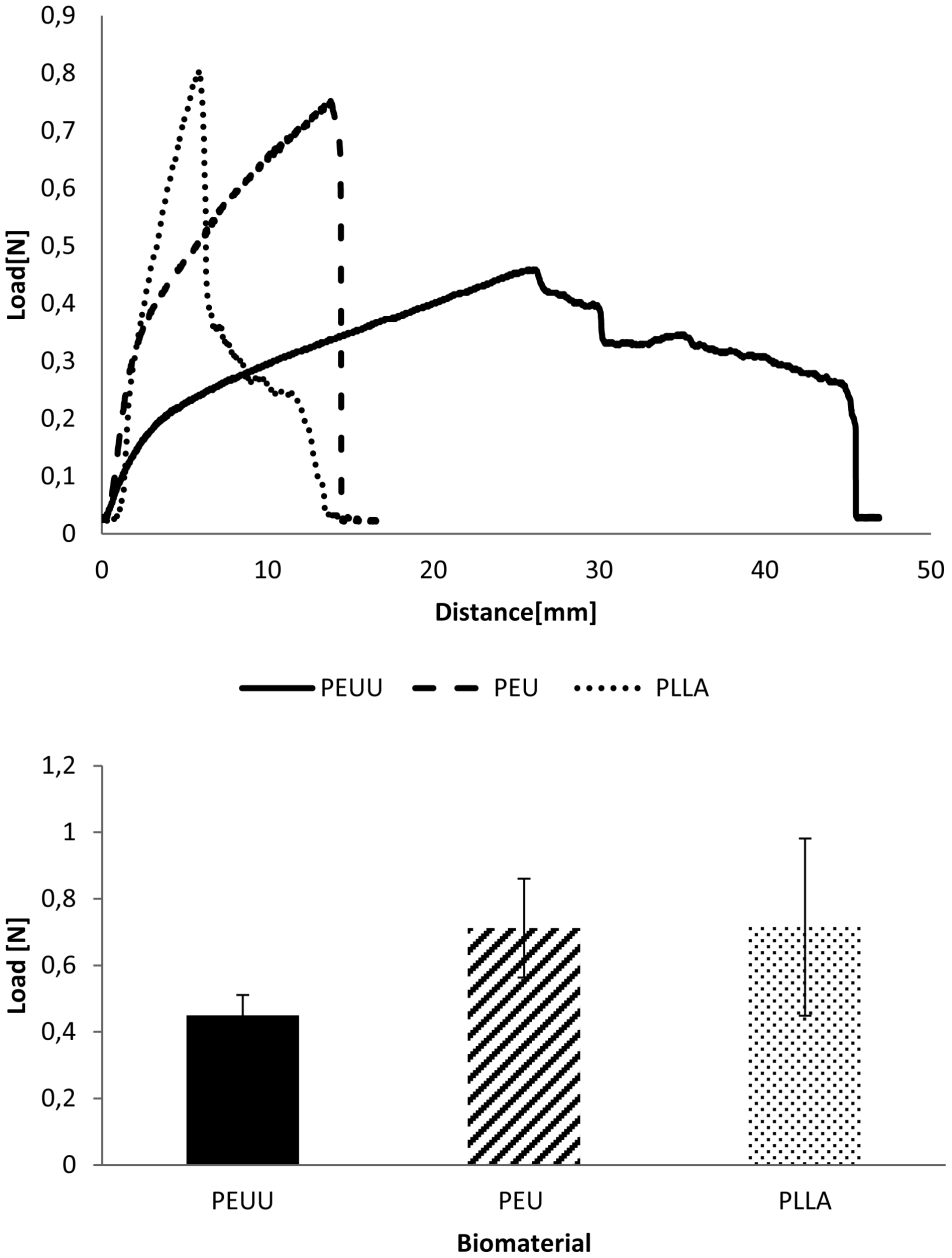
Tensile properties, Tensile strength (A) and tensile strength at break (B) of electrospun fleeces prepared from different polymers.

To test the tensile strength the fleece materials were clamped into a tensile testing machine and stretched until break (Figure 5). For tensile strength the distance until break was measured. PLLA and PEU both expanded about 15 mm whereas PEUU could be lengthened until 45 mm and therefore showed the highest elasticity. Furthermore, the forces at break were measured whereby PEUU reached about 0.45 N, the other materials disrupted at about 0.8 N.

### Degradation Characteristics

**Figure 6:**
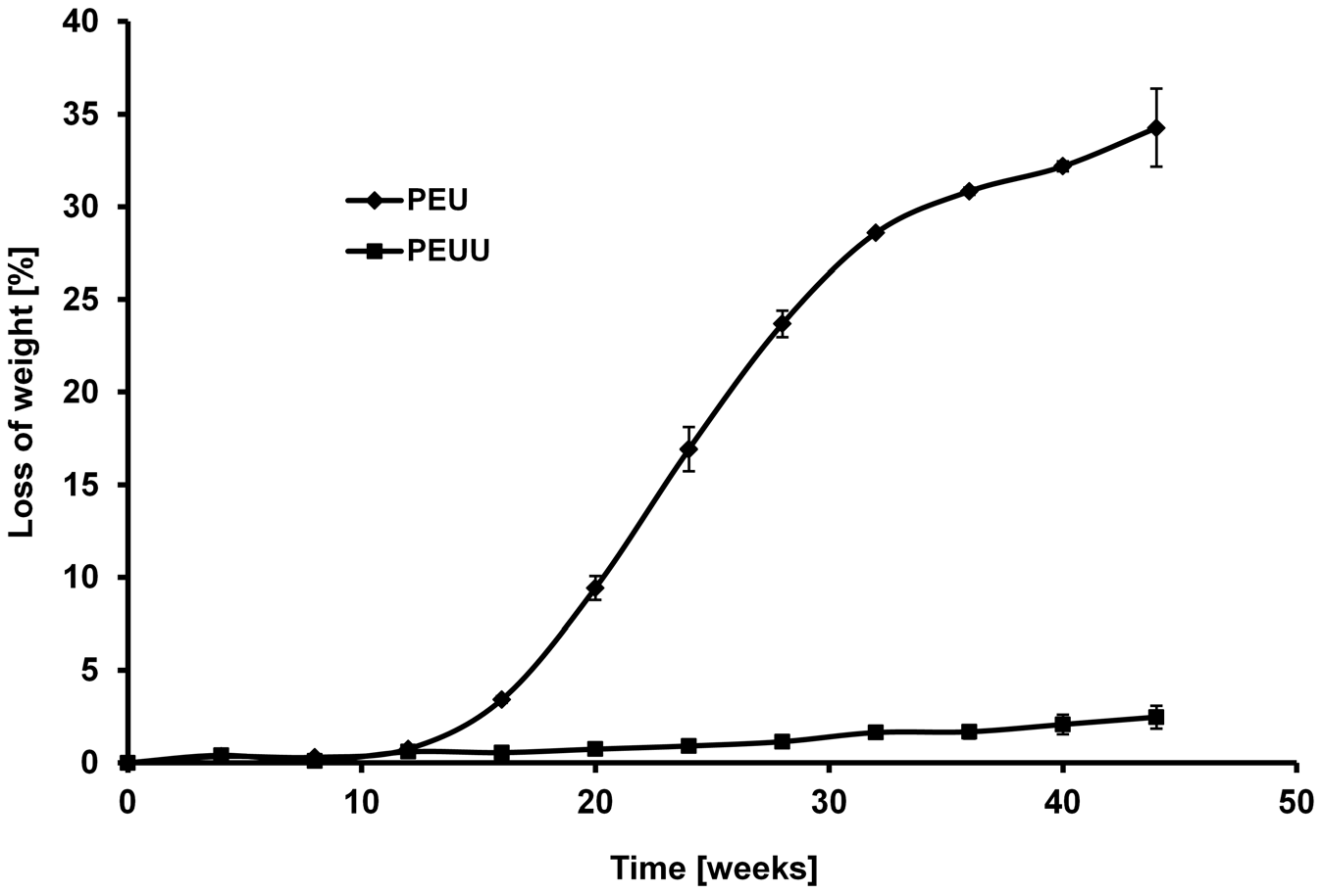
Degradation of compact samples of PEUU and PEU within 44 weeks in Sörensen phosphate buffer at 37°C.

In a first set of experiments compact samples of polymers PEUU and PEU were incubated at 37°C in Sörensen phosphate buffer and the weight loss was determined after defined time periods. After 44 weeks polymer samples show a reduction in weight of 2.4% (PEUU) and 34.3% (PEU), respectively. The polymer with the polylactide-block (PEU) degrades significantly faster (Figure 6).

Due to the low weight of electrospun fleece materials which makes the gravimetric determination of the degradation behaviour difficult we were looking for another analytical methods better suited for electrospun polymer fleeces.

**Figure 7:**
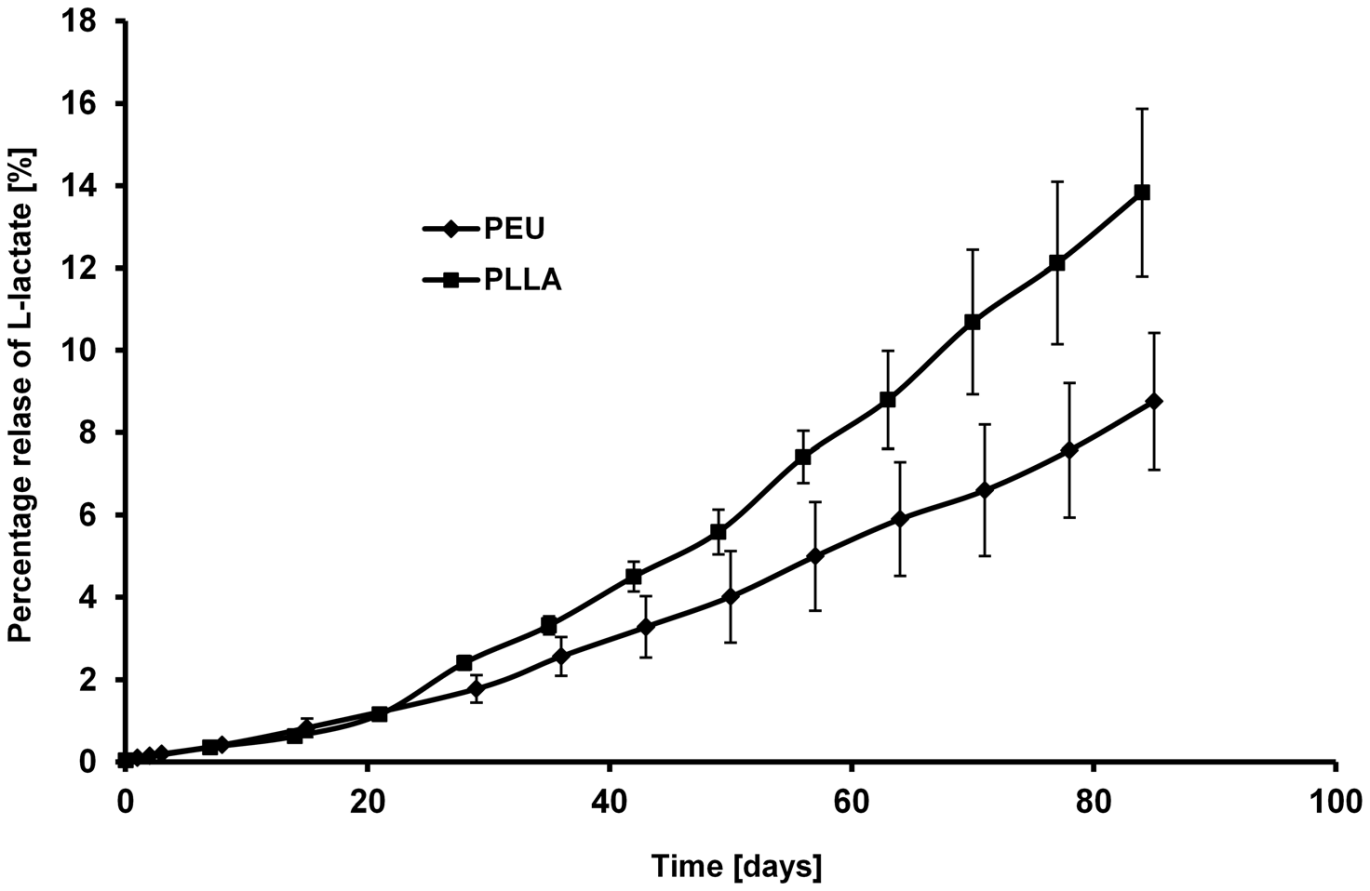
Percentage release of L-lactate from PEU and PLLA (referred to the total amount of L-lacide in non-degraded polymers) during degradation in SBF medium at 37°C.

It is known that the degradation of lactide containing polymers can also be monitored by the measurement of free L-lactate formed via hydrolyis of the polylactide block in the supernatant of the degradation medium. We used this method to study the degradation of lactide-containing PEU and PLLA fleeces. The amount of free L-lactate was measured by an enzymatic assay. At first, the hydrolytic degradation of PEU under strongly elevated degradation conditions (reflux of fleeces for 24 h in 1 N NaOH solution) was investigated to determine the total ( =  initial) amount of releasable L-lactide in the PEU fleece to be 20.7% (w/w). As specified by the manufacturer PLLA contains 85% (w/w) L-lactide ( =  initial amount). Afterwards, degradation of PEU and PLLA was performed at 37°C in SBF medium measuring the amount of released L-lactate under these conditions related to the total L-lactide content of the two polymers. As shown in Figure 7, after 12 weeks, 8.8% of the initial amount of L-lactate from the poly(L-lactide) block of PEU and 13.8% of the initial amount of L-lactate from PLLA were released into the supernatant.

Based on the performed degradation studies the degradation rate of the three polymers during an initial degradation period of about 10 months is in the order PLLA > PEU > PEUU. Long-term studies on the degradation behaviour of PEU and PEUU are in progress now.

### Biological Characterization

#### Cell Viability and Morphology

**Figure 8:**
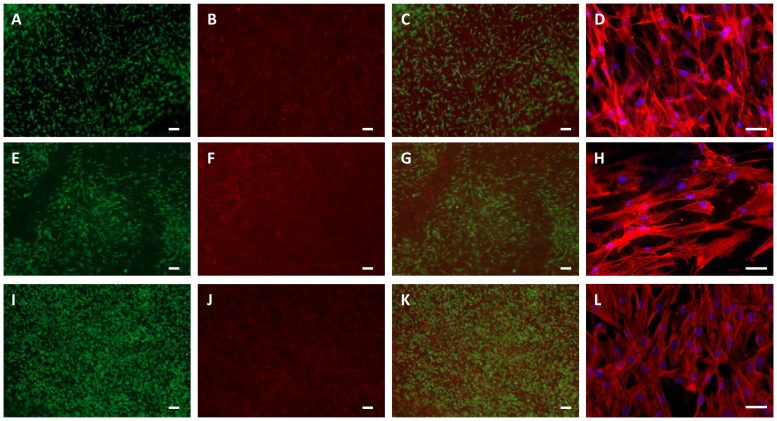
Cell viability and morphology. ASCs were grown on biomaterial PEUU (A-D), PEU (E-H) and PLLA (I-L) for 48 hours. Viability was detected by fluorescein diacetate (living cells; A, E, I) and ethidium-homodimer-1 (nuclei of dead cells; B, F, J) staining. C, G and K are merged pictures. Morphology was detected by TRITC-phalloidin (cytoskeleton, red) and DAPI (nuclei, blue) staining (D, H, L). Bars represent 100 µm (A-C, E-G, I-K) or 50 µm (D, H, L).

Cell viability and cell morphology are depicted in Figure 8. ASCs were cultivated on top of PEUU, PEU and PLLA for 48 hours. Viability was detected by fluorescence staining of cytoplasm of viable cells using fluorescein diacetate and the nuclei of dead cells were stained using ethidium-homodimer-1 (Figure 8). All tested electrospun materials exhibited similar ASCs viabilities. On all three materials only few dead cells could be detected and ASCs show a good viability.

To analyze cell adhesion and morphology, cytoskeleton was stained with TRITC-phalloidin. Actin fibers were well expressed and spread into cell processes. Cells aligned and spread in the direction of the polymers fibers. As already shown by scanning electron microscopy (Figure 4, D-I), ASCs grown on PEUU and PLLA scaffolds had a more spread morphology whereas cells cultivated on PEU were rather elongated.

#### Proliferation of ASCs

**Figure 9:**
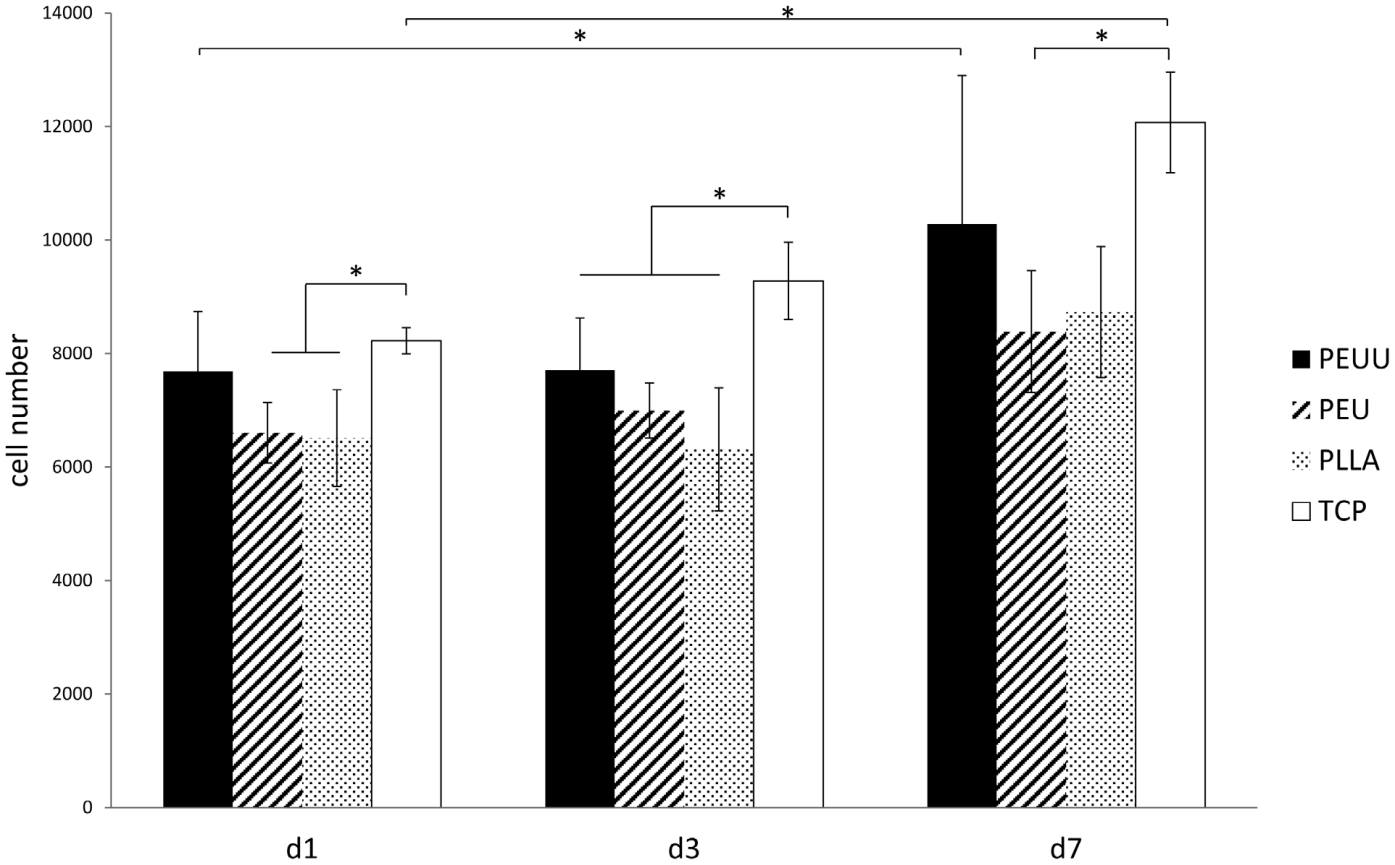
Cell viability of ASCs grown on biomaterials for one, three and seven days was detected by MTT Assay. One representative experiment out of four independent experiments is shown.

Proliferation and viability of ASCs were evaluated by MTT Assay. After 24 hours, viability of cells grown on the materials was comparable to each other. However, after seven days of proliferation, viability of cells grown on PEU and PLLA was by trend higher compared to PEUU (not significant) (Figure 9).

#### Differentiation into the adipogenic Lineage

**Figure 10:**
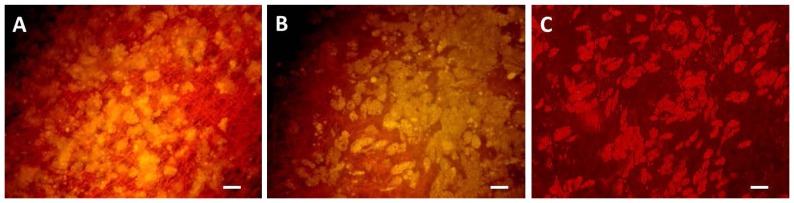
Adipogenic differentiation. ASCs cultivated on biomaterials PEUU (A), PEU (B) and PLLA (C) and differentiated for 21 days into an adipogenic lineage. Cells are stained with AdipoRed to show fat droplets. Bars represent 100 µm.

For determination of differentiation potential ASCs were grown on biomaterials PEUU, PEU and PLLA for two days followed by induction of adipogenesis for three days and further cultivation in nutrition medium. On day 21 lipids were stained with AdipoRed. On all three materials cells were able to be differentiated into the adipogenic lineage as shown by intracellular accumulation of lipid droplets (Figure 10). No striking differences could be observed between the scaffolds.

#### Gene Expression Analysis of Differentiating Cells

**Figure 11:**
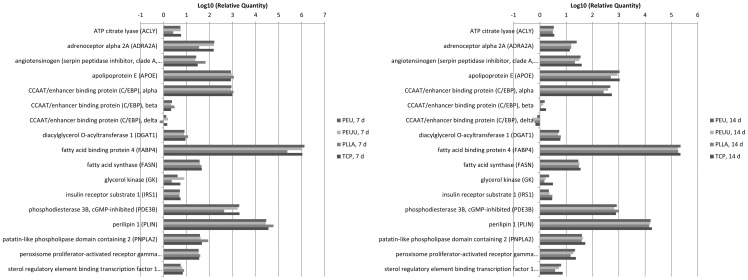
ASCs) were differentiated for seven and fourteen days on polymer-coated plates as well as on typical cell culture plates (TCP). Afterwards, expression of 17 genes associated with adipogenesis and lipogenesis was determined by qRT-PCR. All Ct values were normalized to GAPDH. Mean values of expression levels from 2 different donors are depicted relative to undifferentiated cells set as 1.

Lastly, we sought to gain insights, whether the biomaterials have any effects on the activation or regulation of genes/receptors, which are relevant for the typical behavior of adipocytes and thus are involved in adipogenic, lipolytic or lipogenic processes. To focus on the material effects and to exclude potential effects on the cell behavior caused by 3D culturing conditions, cells were grown in polymer-coated wells (2D condition). ASCs were differentiated for 7 and 14 days into the adipogenic lineage and the expression of 17 genes was determined by qRT-PCR. Results showed that there was no relevant difference in gene expression of the 17 analyzed genes between cells grown on polymer-coated surfaces compared to cells cultivated on the uncoated tissue culture plate or on plates coated with polymer PLLA. None of the observed genes/receptors was significantly affected by the fleece materials (Figure 11).

## Discussion

In our study, poly(L-lactide-co-D,L-lactide) (PLLA) served as control, since this is an established commercially available scaffold for tissue engineering. Degradation of polylactides in the human body via a hydrolytic reaction results in L-lactic acid, a natural intermediate in metabolism [53]. PLLA has been utilized for both in vitro and in vivo studies as 3D scaffolds or grafts for adipose tissue engineering, showing potential in supporting tissue regeneration [54], [55]. Above all, pure poly(L-lactide) and copolymers such as poly(L-lactide-co-D/L-lactide) or poly(D/L-lactide-co-glycolide), are already approved by the Food and Drug Administration for clinical applications [56] and have been shown to provide favorable growth and proliferation conditions for several cell types [57]. An often discussed disadvantage of this polymer type is the formation of hydroxy acids as degradation product which may cause cytocompatibility problems by lowering the pH, especially in the late phase of degradation assuming that the degradation follows a bulk erosion mechanism [58], [59].

Normally, poly-ε-caprolactone (PCL) forms softer polymer materials but shows slower degradation kinetics than PLA [60].

In this study we used ε-caprolactone- and ε-caprolactone-L-lactide containing polyester structural building blocks to construct multiblock polymers in which the polyester segments are linked together by different linking units. In the poly[(L-lactide-co-ε-caprolactone)-co-(L-lysine ethyl ester diisocyanate)-*block*-oligo(ethylene glycol)-urethane] (PEU) polyester segments are linked by urethane bonds whereas in poly(ε-caprolactone-co-urethane-co-urea) (PEUU) both urethane and urea bonds are used to elongate the polyester segments.

The chemical properties of such polymers enable degradation through hydrolytic ester cleavage similar to polylactones like PLA or its copolymers. Emerging degradation products are metabolized and naturally depleted by the surrounding tissue. However, due to the different linkage units in these polymers (ester (-CO-O-), urethane (-NH-CO-O-), and urea (-NH-CO-NH-) bonds) polymer degradation differs from that of PLA with respect to the different hydrolytic sensitivity of the corresponding linkages. Ester bonds are normally cleaved faster than urethane or urea ones. This can be also observed in the case of our polymers. Whereas during the initial degradation period of about 10 months degradation of PEU is only slightly diminished compared to PLLA which is used as a reference, PEUU degrades much slower. Varying the type of linkage between the different polymer segments may therefore represent an effective tool to tune the degradation behavior of scaffold materials. It may be an additional advantage that during the cleavage of urethanes or ureas no carboxylic acids are formed.

It is well known that not only the degradation profile but also the mechanical properties of PCL can be modified by blending or copolymerizing with other polyesters [61]. Its combination with polyurethanes or polyureas led to a higher elasticity of the material as we were able to show in the tensile strength tests. In regard to its use in soft tissue engineering a certain degree of elasticity is mandatory. Our investigations show that PEUU also led to a longer degradation time, which is eligible in soft tissue engineering, since differentiation of ASCs into mature adipose tissue takes several weeks and months. An advantage of the two new polymers is their solubility in volatile organic solvents (acetone, chloroform) well suited for electrospinning. Using this technique fibrous scaffold matrices become available possessing a structure similar to the native extracellular matrix of many tissues. The electrospun scaffolds are characterized by a high interconnective porosity and a high surface-volume ratio facilitating the transport of nutrients and waste products as well as cell adhesion and communication processes. Having this set of properties in mind, electrospun scaffolds of PEU and PEUU may also be of interest for other soft tissue engineering applications like nerve injury repair, tendon/ligament reconstruction or vascular tissue engineering.

We could show that cell compatibility with adipose-derived stem cells was good as ASCs were able to adhere and proliferate on both new scaffolds. Morphology of the cells was slightly better on PEUU than on PEU showing a more physiological appearance. ASCs were also able to differentiate on both scaffolds which is mandatory for adipose tissue engineering. Cells were forming lipid droplets showing a physiological phenotypical appearance after 21 days. In addition gene analysis of 17 genes involved in adipocytes differentiation and lipid storage revealed no unwanted effects of the new materials on gene expression of differentiating ASCs. These include the peroxisome proliferator-activated receptor gamma (PPARgamma) known to be a master regulator of adipogenesis [62], [63] and members of the C/EBP protein family also transactivating adipocytes specific genes [64]. Also perilipin A, which is supposed to prevent lipase access and therefore is important for the lipid storage of mature adipocytes [65] and the fatty acid binding protein (FABP4), which also contributes to the acquisition of the adipocytes phenotype [66] were expressed in a typical way.

## Conclusion

In accordance with our initial hypothesis we found that the electrospun scaffold materials based on the two newly synthesized polymers PEU and PEUU cover a broad range of properties due to their different chemical composition and structure. Especially with regard to their mechanical characteristics and their in vitro degradation behaviour the materials strongly differ from each other. PEUU electrospun scaffolds showed higher elasticity, which in our opinion is favorable in terms of soft tissue engineering. Concerning degradation the PEU scaffold disintegrated significantly faster in vitro than the PEUU material. ASCs were able to adhere, proliferate and differentiate on both polymeric scaffolds. Overall, we can conclude that both PEU and PEUU meshes can serve as a useful scaffold for adipose-derived stem cells and tissue engineering. Further investigations have to follow to verify the suitability of the tissue substitute in vivo.
